# Sidedness in Unilateral Orofacial Clefts: A Systematic Scoping Review

**DOI:** 10.1177/10556656231221027

**Published:** 2023-12-13

**Authors:** Matthew Fell, Daniel Bradley, Ambika Chadha, Sophie Butterworth, Amy Davies, Craig Russell, Bruce Richard, Yvonne Wren, Sarah Lewis, David Chong

**Affiliations:** 1 Spires Cleft Centre, John Radcliffe Hospital, Oxford, UK; 2 The Cleft Collective, Bristol Dental School, 1980 University of Bristol, Bristol, UK; 3 20313 Operation Smile Inc, Virginia Beach, Virginia, USA; 4 Cleft.Net.East, University of Cambridge NHS Hospitals Trust, Cambridge, UK; 5 Department of Perinatal Imaging and Health, Kings College London & South Thames Cleft Service, St. Thomas Hospital, London, UK; 6 Cleft Registry and Audit Network, Clinical Excellence Unit, 14211 The Royal College of Surgeons of England, London, UK; 7 The Cleft Collective, Bristol Dental School, 1980 University of Bristol, Bristol, UK; 8 59842 Cleft Care Scotland, Royal Hospital for Children, Queen Elizabeth University Hospital, Glasgow, UK; 9 West Midlands Cleft Service, Birmingham Women and Children's Hospital, Birmingham, UK; 10 The Cleft Collective, Bristol Dental School, 1980 University of Bristol, Bristol, UK; 11 Speech and language therapy research unit, North Bristol NHS Trust, Bristol, UK; 12 The Cleft Collective, Bristol Dental School, 1980 University of Bristol, UK; 13 Plastic and Maxillofacial Surgery, The Royal Children's Hospital, Melbourne, Australia

**Keywords:** cleft lip, cleft lip and palate, etiology, genetics, outcomes

## Abstract

**Objective:**

An overview of the literature relating to the sidedness of unilateral cleft lip with or without cleft palate to map current knowledge on the cause and impact of directional asymmetry.

**Design:**

Scoping review with a systematic search of Medline and Embase from inception to May 2023.

**Patients, Participants:**

Humans born with a left or right unilateral cleft lip with or without a cleft palate.

**Main Outcome Measures:**

Cleft sidedness as a co-occurrence, an outcome or an exposure.

**Results:**

Forty studies were eligible for inclusion and confirmed the predilection for the occurrence of left sided cleft lips; 12 studies reported cleft sidedness co-occurring with another phenotype, 11 studies report sidedness as an outcome and 17 studies as an exposure. Phenotypes which were reported to co-occur with either left or right sided clefts included congenital dental anomalies, handedness and additional congenital anomalies. Variables investigated as a potential cause of left or right sided clefts as an outcome included chromosomal anomalies, genetic variants and environmental factors. Outcomes investigated in relation to cleft sidedness as an exposure included facial anatomical features, facial growth, educational attainment, functional and psychological characteristics. More studies showed worse outcomes in right sided clefts versus left sided clefts than vice versa, although studies were inconsistent, and a quality assessment was not performed.

**Conclusions:**

The field of cleft sidedness research is expanding and there are promising early findings to differentiate cause and outcome by sidedness of the cleft.

## Introduction

The prevalence of left sided unilateral cleft lip with or without cleft palate (UCL/P) is consistently observed to be twice that of right sided UCL/P across multiple studies and populations.^1^

Lateralization to left or right is one of the three major axes of polarisation during embryological development, together with anterior/posterior and dorsal/ventral orientations. The developmental timing of lateralization in the human body occurs after the other two axes of polarization have occurred, shortly after gastrulation at the fourth week of gestation.^2^ Whilst most aspects of the body are symmetrical about the sagittal plane, there is asymmetry of the organs in the thoracic and abdominal regions and hemispheric specialisation in the brain.^3^ The asymmetry of body organs is driven by a complex cascade of molecular pathways including Nodal and the transcription factor Pitx2.^4^

The embryological primary palate (upper lip and alveolus up to the incisive foramen) is formed from the fusion of medial nasal and maxillary processes between 4–7 weeks of gestation, which coincides with the timing of organogenesis.^5^ Clefts of the embryological primary palate most commonly occur as a left unilateral cleft lip, a right unilateral cleft lip or a bilateral cleft lip in a ratio of 6:3:1.^6,7^ A unilateral cleft of the lip is characterised by a gap in the upper lip through the left or right philtrum to the underlying alveolus, between the ipsilateral lateral incisor and canine tooth, following the line of suture incisiva up to the foramen incisivum.

The embryological secondary palate (hard and soft palate posterior to the incisive foramen) develops later between 8–12 weeks of gestation, from paired outgrowths on the maxillary processes called palatal shelves. The palatal shelves are initially positioned vertically and subsequently reorientate into the horizontal plane prior to midline fusion, a process which is often asynchronous with one shelf reorientating before the other.^8^ A unilateral cleft of the lip and palate (UCLP) is characterised by a lateralized cleft within the hard palate, with fusion of the vomer to the non-cleft palatal shelf, whereas the cleft of the soft palate is a midline defect, with the musculature of the velum and uvula failing to unite. This phenomenon is illustrated in the LAHSHAL classification^9^ where clefts of the lip, alveolus and hard palate are coded twice to enable documentation of involvement of a left or a right side, compared to the midline cleft of the soft palate which is coded only once.

Our aim was to gain an understanding of the current knowledge base on the cause of directional asymmetry in UCL/P and its impact on outcomes. Scoping reviews are characterized by a systematic approach to map evidence and are well suited to summarizing bodies of heterogenous literature, identifying research gaps and making recommendations for further research.^10^ The key difference from a systematic review is that a scoping review provides an overview of the literature regardless of quality, therefore a quantitative synthesis or meta-analysis is not undertaken.^11^

Our objectives in this scoping review were threefold: firstly, to systematically search the published literature for studies which have reported on sidedness in UCL/P, secondly to assess what is known about the determinants of sidedness in UCL/P via embryological, genetic or environmental processes and thirdly to determine whether there are any differences in outcomes reported when stratified by sidedness of UCL/P.

## Methods

### Protocol and Registration

This study followed the Joanna Briggs Institute guidance for conducting a systematic scoping review^10^ and was reported according to PRISMA-ScR guidance^11^ (see supplementary Table 1). A full protocol of this review is available from the PROSPERO systematic review register (registration number CRD42022333191: https://www.crd.york.ac.uk/prospero/display_record.php?RecordID = 333191).

**Table 1.** Included Studies Reporting Left Versus Right Sided UCL/P as a co-Occurrence.

Main Variable Category	Author and year	Origin of participants	Study Design	Cleft subtype	Left clefts	Right clefts	Variable measure	Adjusted Confounders	Reported Results
**Congenital dental anomalies**	Letra et al., 2007^12^	Brazil	Case control	UCL/P	172	78	Clinical assessment of permanent dentition and radiographs.	Nil	Tooth agenesis was more frequent on the right maxilla in left UCL/P (5/78 vs 16/172; p = 0.01) and was more frequent on the left maxilla in right UCL/P (8/78 vs 6/172; p = 0.01).
	Akcam et al., 2010^13^	Turkey	Case series	UCLP	53	34	Radiographs, dental casts and intra-oral photographs of permanent dentition.	Nil	Tooth agenesis was more frequent on left maxilla in left UCLP compared to right UCLP (84/53 vs 22/34; P < 0.001).
	Kuchler et al., 2011^14^	Brazil	Case series	UCLP and UCL + -A	71	33	Clinical orthodontic records, dental casts and radiographs of permanent dentition.	Nil	Tooth agenesis was similar in right CL/P (12/33) and left CL/P 22/71)
	Matern et al., 2012^15^	France	Case series	UCLP and UCL + -A	45	15	Clinical assessment and radiographs of permanent dentition.	Nil	Tooth agenesis was more common on the left side for both left CL/P (58.5%) and right CL/P (66.7%).
	Eslami et al., 2013^16^	Iran	Cross sectional	UCL/P	32	25	Intra-oral photographs, dental casts and radiographs of permanent dentition.	Nil	Contralateral lateral incisor agenesis was more common in RUCL/P than left UCL/P (5/25 vs 0/32; p = 0.015)
	Mangione et al., 2018^17^	France	Case series	UCLP	25	12	Radiographs of permanent dentition.	Nil	Homolateral lateral incisor agenesis was greater for left compared to right UCLP (16/25 vs 5/12; p < 0.001) as was contralateral lateral incisor agenesis (9/25 vs 5/12; p < 0.05)
**Handedness**	Rintala 1985^18^	Finland	Case series	UCL/P	220	128	Self-reported questionnaire and observation of handedness	Nil	Proportion of left handedness was greater for left CL/P (32/220) than right CL/P (6/128)
	Yorita and Melnick 1988^19^	USA	Case series	UCL/P	93	56	Self-reported questionnaire	Sex	Proportion of left handedness was greater for left CL/P (41/93) than right CL/P (5/56) independent of sex
	Jeffery and Boorman 2000^20^	United Kingdom	Cross sectional	UCL/P	105	70	Parental questionnaire	Nil	Proportion of left handedness was similar for left CL/P (16/105) and right CL/P (11/70)
	Daskalogiannakis et al., 1998^21^	Canada	Cohort	UCL/P	198	91	Self-reported questionnaire	Nil	Proportion of left handedness was greater for left CL/P (22/198) than right CL/P (4/91)
**Additional congenital malformations**	Gallagher et al., 2018^22^	USA	Case series	UCL + -A and UCLP	276	155	Additional congenital malformations identified from medical records from single institution.	Age, sex, median income, language, Medicaid status and adoption status	With left UCL + -A as a reference there was a similar risk of additional malformations in right CL + -A (OR 1, 95%CI 0.3–3.1) but a greater risk of malformations in both left UCLP (OR 2.3 95%CI 1.1–5.1) and right UCLP (OR 3.5 95%CI 1.5–7.9).
	Fitzsimons et al., 2022^23^	United Kingdom	Cohort	UCLP and UCL + -A	2406	1379	National hospital admission records linked to national cleft registry.	Nil	Proportion of additional congenital malformations similar between left and right UCL + -A (21% vs 22%) but there was a difference in the proportion of malformations in left compared to right UCLP (23% vs 32%)
	Ogawa et al., 2023^24^	Japan	Case series	UCL/P	58	35	Clinical records in hospital	Nil	Proportion of additional congenital malformations less in left compared to right UCL/P (19% vs 40%; p < 0.01)

### Identification of Studies

Eligible studies were defined as full-text publications reporting on humans born with UCL/P. The protocol included descriptive and analytical study designs and both primary and secondary data. We included case series and case reports, but commentaries or editorials were not included (see Supplementary Table 2 for inclusion and exclusion criteria).

The variable of interest was unilateral cleft sidedness, defined as a left or right sided UCL/P. We focused on the sidedness of unilateral cleft lip, rather than the sidedness of clefts in the alveolus or hard palate because of the external nature of the lip, making it easier and more reliable to determine left versus right. Studies were categorized depending on whether they considered UCL/P sidedness as a co-occurrence, an outcome or an exposure. Where UCL/P sidedness was a co-occurrence, studies reported the prevalence of additional phenotypes. Where UCL/P sidedness was an outcome, studies reported variables involved in the aetiology of a cleft. Where UCL/P sidedness was an exposure, studies reported anatomical and functional features.

The databases searched included Medline and Embase from inception to 22^nd^ May 2023. The search was tailored individually to each database with input from a University Librarian (see Supplementary Table 3 for search strategies) and there were no language restrictions. The search focused on published literature and did not include grey literature. In addition, manual searches of reference lists of all studies included in the review were performed.

Titles and abstracts were reviewed independently in the first round of screening by two reviewers (MF/DB) according to the specified inclusion and exclusion criteria. Differences were resolved through discussion to reach a consensus. In the second round of screening, full text screening was performed independently by the same two reviewers for inclusion and any disagreements resolved through discussion. When multiple reports of a single study were identified, the report with the greatest number of patients was selected. The Rayyan web application was used to facilitate the screening process.^25^

### Data Extraction and Synthesis

Data was extracted via Microsoft Forms into an Excel spreadsheet. Data extracted included: title, authors, publication year, country of study population, study design, sample size, confounding factors and reported results. Due to the potential for studies to report multiple outcomes and exposures, we reported all pertaining to UCL/P sidedness for transparency, to avoid selecting only positive results. Due to the heterogeneity of study designs it was not possible to perform a meta-analysis, so results were reported in tables. A descriptive summary and narrative synthesis of the included studies was performed in accordance with published guidance.^26^

## Results

### Study Selection and Study Characteristics

**Figure 1.**
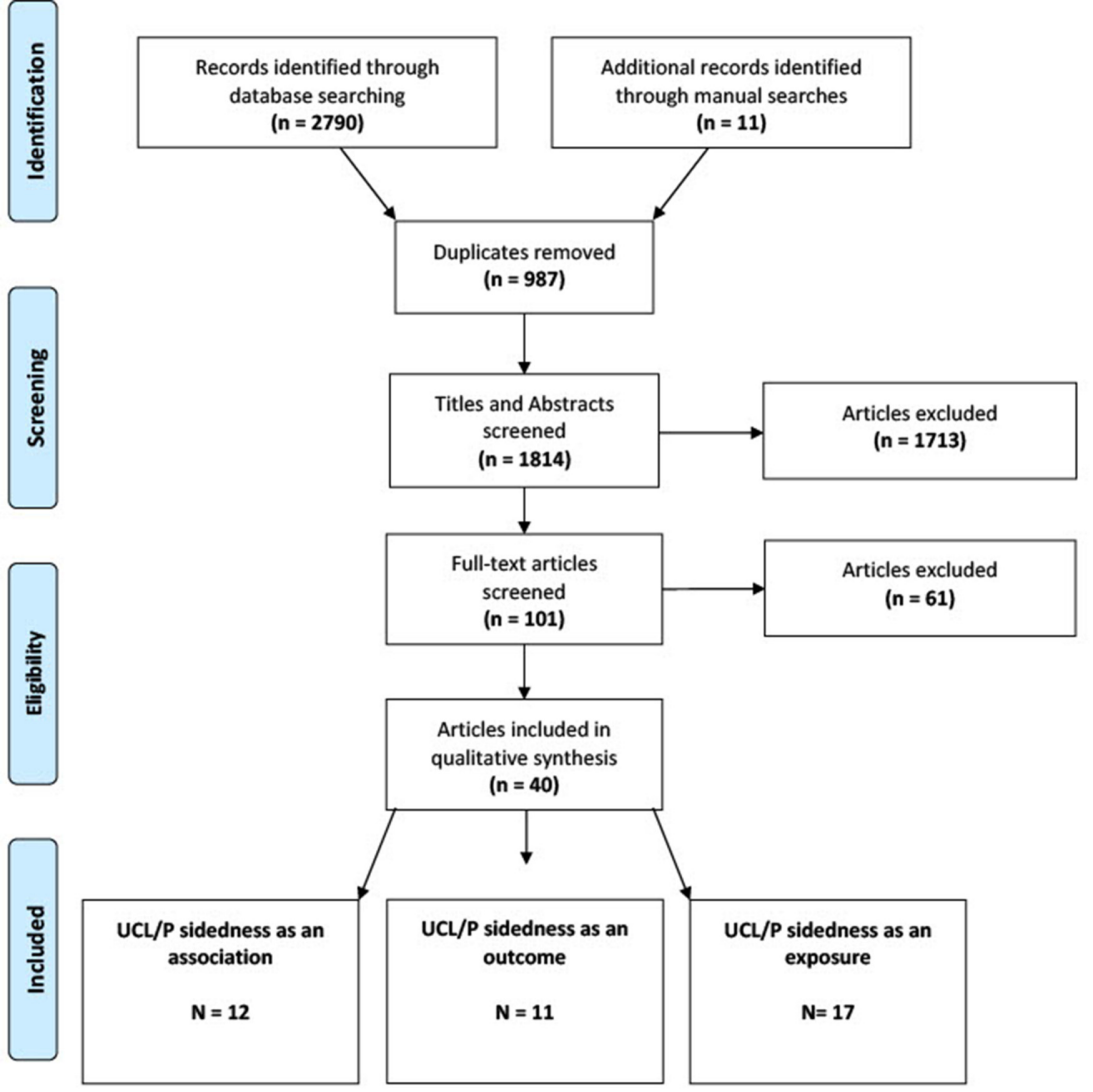
A flow chart of the search strategy and study selection.

A flowchart for the review process is shown in Figure 1. A total of 2790 citation records were identified from searching the two databases and a manual search of included articles identified 11 additional studies. After exclusions, 40 studies were included in the scoping review. The earliest study to be included in the review was published in 1985.^18^ Thirty nine studies were published in the English language and one study was published in German.^27^ Participants in the studies were reported to have the following unilateral cleft lip sub phenotypes: unilateral cleft lip with or without cleft palate (UCL/P: n = 16), unilateral cleft lip and palate (UCLP: n = 14), unilateral cleft lip with or without a cleft of the alveolus (UCL + -A n = 4) and finally distinct populations of both UCLP and UCL + -A (n = 16).

Twelve studies reported UCL/P sidedness as a co-occurrence (Table 1), 11 studies reported UCL/P sidedness as an outcome (Table 2) and 17 studies reported UCL/P sidedness as an exposure (Table 3). In total, 9104 left sided UCL/P (66%) and 4890 right sided UCL/P (34%) were reported from the 40 studies, and whilst we cannot exclude patient overlap, this does align with the anticipated predominance of left sided UCL/P.

**Table 2.** Included Studies Reporting Left Versus Right UCL/P as an Outcome.

Main exposure Category	Author and year	Origin of participants	Study Design	Cleft subtype	Left clefts	Right clefts	Variable measure	Adjusted Confounders	Reported Results
**Chromosomal anomalies**	Masuzaki et al., 2003^28^	Japan	Case report	UCL + -A	1	1	Chromosomal analysis in mono-zygotic twins and PCR genotyping of 50 loci	Nil	Discordant sidedness of cleft despite identical alleles at 50 loci examined
	Takahashi et al., 2018^29^	Japan	Case report	UCLP	1	1	Whole-genome sequencing from saliva samples in monozygotic twins	Nil	Discordant sidedness of cleft despite no discordant DNA variants identified
**Genetic variant**	Farina et al., 2002^30^	Italian ancestry	Case-parent -trio	UCL/P	31	32	Linkage to chromosome 6 using pattern and latent class analysis	Nil	Right UCL/P were more likely (9 out of 10 cases) to show linkage to chromosome 6 than left UCL/P (3/10; p = 0.02)
	Letra et al., 2009^31^	Caucasian from Brazil	Case -Control	UCL/P	140	60	PCR genotyping of genetic markers in AXIN2 and CDH1	Nil	Genetic markers in AXIN2 and CDH1 examined for associations with cleft subtypes but did not do a formal comparison of the difference between left and right-sided clefts.
	Carlson et al., 2017^32^	Asian (Chinese and Filipino)	Case-parent- trio	UCL/P	459	259	Common and rare variant analysis using sequence data from 13 cleft associated genetic regions	Nil	No strong evidence for association between right and left UCL/P and common variants but differences observed with 13 rare variants in the FGFR2 gene.
	Yin et al., 2021^33^	Han Chinese	Case Control	UCL + -A	480	253	5 SNPs across GREM1 and TP63 which were found to be associated with all non-syndromic clefts in subtypes of cleft	Nil	SNP rs1345186 in the TP63 gene was associated with right CL + -A but no formal comparison of the difference between left and right sides.
	Curtis et al., 2021^34^	Multi-ethnic cohort from USA	Cohort	UCL + -A + UCLP	852	446	Subtype specific GWAS followed by a modifier analysis of SNPs from the GWAS, reaching a p-value <1 × 10^−5^ in either left or right sided clefts	Nil	Region on 4q28 downstream from FAT4 gene approached significance in left UCL + -A vs right UCL + -A. Different effect sizes in UCL compared to UCLP
	Tao et al., 2022^35^	Han Chinese	Case Control	UCL + -A	480	253	3 SNPs in the NTN1 gene for associations with cleft subtypes	Nil	The study investigated left and right UCL + -A as separate subtypes but did not compare the two. G allele at rs9891446 associated with right (OR = 1.44, 95% CI: 1.1–1.88; P = 0.0073).
**Environmental factors**	Carinci et al., 2005^36^	Southern Italy	Cohort	UCL/P	47	20	Univariate analysis of association with sex, smoking, folic acid, diabetes	Nil	Right UCL/P more common in females (p = 0.0281), left UCL/P more common in males (p = 0.0359). Association of smoking, folic acid and diabetes with cleft sidedness unclear.
	Hermann et al., 2018^37^	Denmark	Cohort	UCL/P	230	123	Univariate analysis of parental age	Nil	No association between parental age and right or left sided CL/P
	Kruse et al., 2021^38^	Caucasian from Germany	Cohort	UCL/P	394	193	Interviews to ascertain cigarette smoking in first trimester	Family history, folic acid, maternal age and alcohol use.	There was weak evidence that children of smoking mothers more often had right CL/P than children of non-smoking mothers (42% versus 31%; p = 0.041).

**Table 3.** Included Studies Reporting Left Versus Right Sided UCL/P as an Exposure.

Main Variable category	Author and year	Country of participants	Study Design	Cleft Type	Left Clefts	Right Clefts	Outcome measure	Adjusted Confounders	Reported Results
**Anatomical differences**	Filho et al., 1994^39^	Brazil	Case series	UCLP	1313	701	Pre-operative presence of Simonart's Band in infancy	Nil	Presence of Simonart's band similar between left (19.3%) and right (20.1%) UCLP
	Ras et al., 1994^40^	Netherlands	Case control	UCLP	32	17	Facial asymmetry determined by 3D coordinates at mean 7 years of age	Sex and age	Values were not reported but figures showed left side of face was larger in vertical and sagittal plane in right CLP whereas right side of face was larger in vertical and sagittal planes in left CLP
	Feragen et al., 1999^41^	Norway	Case series	UCLP	30	30	Psychology students rating perceived disfigurement of 2D photos using visual analogue scales	Nil	Faces of left UCLP less disfigured than right UCLP (F = 224.26; p < 0.001). No difference in outcome between both normal and mirror reversed photos (F = 0.03; p > 0.05).
	Van der Plas et al., 2010^42^	USA	Case control	UCL/P	19	14	Volume of male brain regions on MRI	Age and intracranial volume	Total white matter reduced in both hemispheres in right UCL/P (mean 440.1 cm^3^) compared to left UCL/P (463.5 cm^3^) and healthy controls (461.3 cm^3^; p = 0.02). No difference in intracranial volume, CSF volume and gray matter.
	Mulliken and LaBrie 2012^43^	USA	Case series	UCL/P	68	31	Anthropometric measurements of lip at primary cheiloplasty in infancy and repeated at 6 years	Nil	No strong evidence of a percentage change in anthropometric measures of lip in right and left UCL/P pre-op and at 6 years of age.
	Celikoglu et al., 2014^44^	Turkey	Case control	UCLP	22	8	Airway volume on Cone Beam Computed Tomography (CT)	Nil	No strong evidence of a difference in nasopharyngeal airway or oropharyngeal airway volumes in right compared to left UCLP.
	Bella et al., 2016^45^	UK	Case series	UCLP	51	25	Aesthetic outcome of 2D photos via rating scale and Symnose (semi-objective measure of symmetry)	Nil	Left sided UCLP had more favourable Likert scores than right sided UCLP (p = 0.02). Symnose analysis did not show any difference in lip (p = 0.66) or nose (p = 0.69) mismatch between left and right UCLP
	Chong et al., 2022^46^	Canada	Case series	UCL/P	139	80	Anthropometric measurements of lip at primary cheiloplasty in infancy	Nil	Vertical lip height on the lateral lip element more deficient on right CL/P compared to left CL/P (Ratio of cleft to non-cleft measures 0.67 vs 0.72; p < 0.001). Medial lip element height, lateral lip length and vermillion height had similar ratios between right and left CL/P.
**Facial growth**	Haque et al., 2017^47^	Bangladesh	Case series	UCLP	51	33	Dental Arch Relations before orthodontic treatment using GOSLON Yardstick	Age, gender, family history, palatoplasty technique, cheiloplasty technique	No strong evidence for difference in GOSLON scores between right and left sided UCLP (OR 0.215 95% CI 0.045–1.023; p = 0.053)
	Eslamian et al., 2019^48^	Iran	Cross sectional	UCLP	27	18	Cephalometric measurements before orthodontic treatment	Nil	No strong evidence for a difference in 24/28 cephalometric measures between right and left CLP. U1-SN angle (axial inclination labial maxillary central incisor in relation to cranial base) was greater in left CLP (89.2 + - 7.9) compared to right CLP (81.3 + - 10.4). Go-Gn and Go-Pog (both indicating mandibular length) were greater in left CLP (73.8 + - 7.5 mm / 74.1 + - 8.0 mm) than right CLP (68.1 + - 4.5 mm/68.4 + - 4.5 mm).
	Pucciarelli et al., 2021^49^	Brazil	Case series	UCLP	26	9	Dental arch symmetry after orthodontic treatment using dental casts and analysis software	Nil	Left CLP had symmetry in canine-molar and incisal-molar distance whereas right CLP showed symmetry in incisal-canine distance. Overall right CLP had the strongest correlation with dental arch symmetry
	Staudt et al., 2021^50^	Switzerland	Case series	UCLP	46	10	Dental arch relations after orthodontic treatment using dental casts and Modified Bodenham Huddart (MHB) index	Treatment centre, sex, orthognathic surgery	MHB was more positive in left UCLP compared to right UCLP. There was a more marked labial difference in left UCLP compared to right UCLP and the maximum overjet was 0.5 mm larger for left UCLP (p = 0.008). But 65% left UCLP underwent orthognathic compared to 20% right UCLP.
**Educational achievement**	Gallagher et al., 2017^51^	USA	Case control	UCL/P	292	103	Educational achievement using standardized test data in reading, language and mathematics	Grade level, sociodemographic status	Children with left CL/P had lower educational achievement in reading compared to those with right CL/P (Regression coefficients (Standard error) for reading -6.62(3.30; P < 0.05), language -3.40 (3.32; P > 0.05) and mathematics -1.10 (3.01; P > 0.05)).
	Gallagher et al., 2018^22^	USA	Case series	UCL/P	276	155	Need for academic support at school in the form or an individualized educational plan (IEP)	Sex, Medicaid status, median income, language spoken at home, adoption status	Using Left CL + -A as a reference there was weak evidence of difference in educational support for right CL + -A (OR 1.7 95%CI 0.6–4.8) or left CLP (OR2.0 95%CI 0.8–4.5). There was stronger to show a greater need for educational support for right CLP (OR 4.4 95% CI 1.9–10.3)
	Gjerdevik et al., 2021^52^	Norway	Cohort	UCLP and UCL + -A	Not stated	Not stated	Grade point average from middle school graduation at age 16 years	Sex, maternal age, parity, mothers relationship status, parents highest level of education	No values reported but figure in supplementary analysis shows no strong evidence of differences in means for grade point average between right and left UCL + -A and UCLP at 16 years of age.
**Functional**	Dames et al., 2009^27^	Germany	Case control	UCLP	32	23	Speech intelligibility using word recognition rate	Nil	No strong evidence of differences in speech intelligibility between left (Word recognition 49.9% (SD 17.1)) and right UCLP (Word recognition 48.2% (SD 14.6))
	Ogawa et al., 2023^24^	Japan	Case series	UCL/P	58	35	Feeding via NG tube	Nil	The proportion of nasogastric tube feeding was lower for left compared to right UCL/P (27.3% vs 64.3%; P < 0.01)
**Psychological**	Ramstad et al., 1995^47^	Norway	Case control	UCLP	126	45	Self-reported questionnaire on psychological and social wellbeing and adjustment	Nil	No difference in depression or anxiety between right and left UCLP. Right UCLP wanted more surgical treatment than left UCLP (38% vs 14%) but no difference in requests for speech or dental therapy interventions

### UCL/P Sidedness as a co-Occurrence

There were six studies reporting the presence of congenital anomalies of permanent dentition. The studies were heterogenous in terms of dental anomaly classification and inclusion of dental anomalies adjacent to the cleft in the analysis. Tooth agenesis was the anomaly with the highest frequency and was reported in all six studies. A higher frequency of tooth agenesis in left compared to right UCLP was reported in three studies^12,13,17^ whereas one study found no difference in tooth agenesis between left or right UCLP.^14^ Two studies reported right UCL/P being more likely to have contralateral incisor agenesis^15,16^ and Matern et al. (2012) proposed a theory that this could be due to right UCL/P being a lesser form of bilateral CL/P.

Four studies reported on the co-occurrence of handedness with UCL/P sidedness. The proportion of left handedness was greater in left UCL/P compared to right UCL/P in three studies^18,19,21^ whereas another study found no difference in handedness between left or right UCL/P.^20^

Additional congenital anomalies were found to be less prevalent for left compared to right UCL/P in the three studies publishing on this variable.^22–24^ When Fitzsimons et al. and Gallagher et al. stratified by cleft subtypes,^22,23^ they found this trend was true only for UCLP and not for UCL + -A.

### UCL/P Sidedness as an Outcome

Two case reports from Japan performed chromosomal analysis in pairs of monozygotic twins born with discordant sides of UCL/P.^28,29^ Masuzaki et al. reported identical alleles across the 50 sites investigated in the twins and Takahashi et al., reported no differences between the twins following whole genome sequencing despite the different UCLP sidedness profile.

Four candidate gene studies investigated whether specific genetic loci were associated with left and right UCL/P subgroups. These loci investigated were chromosome 6,^30^ TP63 gene on chromosome 3,^33^ CDH1 gene on chromosome 16^31^ and NTN1 gene on chromosome 17.^35^ Only the study by Farina et al.,^30^ of chromosome 6 compared left with right UCL/P, whereas the other studies reported comparisons of sidedness subgroups with controls. In addition to the candidate gene studies, Carlson et al.,^32^ investigated common and rare variants, using sequencing data from 13 cleft associated regions, to determine whether they influenced left versus right UCL/P; they found evidence for 13 rare variants in the FGFR2 gene. Curtis et al.,^34^ conducted subtype specific GWAS then ran a modifier analysis of SNPs from the GWAS, which reached a p-value <1 × 10-5 in either left or right UCL/P to test for differences between the two. They reported that a region downstream of the FAT4 gene on chromosome 4 approached significance in an analysis of left versus right sided UCL/P, raising the potential for distinct genetic modifiers for sidedness.

Environmental factors influencing cleft laterality were investigated in three studies. Maternal cigarette smoking was associated with right UCL/P compared to left UCL/P in a 2021 German cohort,^38^ whereas the relationship of smoking to sidedness was unclear in a smaller Italian cohort.^36^ Hermann et al.,^37^ reported no association with parental age and UCL/P sidedness.

### UCL/P Sidedness as an Exposure

Studies reporting anatomical differences according to UCL/P sidedness covered a broad variety of outcome measurement including anthropometric measurements of the face, perceptive facial attractiveness, brain volumes and airway volumes. Four studies reported anatomical differences of the lip with right UCL/P more often associated with; hypoplasia of the lateral lip element,^46^ more pronounced disfigurement^41,45^ and asymmetry^40^ compared to left UCL/P. Ras et al.,^40^ found the non-cleft side of the face to be asymmetrically larger than the cleft side of the face in the vertical and sagittal planes in both left and right UCLP. Two studies of facial anatomy reported no differences between right and left UCL/P.^39,43^ It is worth noting that the anthropometric measurements were not consistent across studies, therefore direct comparison across studies is not possible. White matter brain volume was reported to be lower for males with right UCL/P compared to males with left UCL/P.^42^ Volumetric analysis of airway anatomy reported no difference between left and right sided UCLP.^44^

Facial growth was measured using cephalometric measurements, arch symmetry assessment and dental arch relationship analysis. A study investigating cephalometric measurement of facial growth reported no difference in most of the 28 measurements reported between left and right UCLP but within the four measurements noted to be different there was greater mandibular length in left compared to right UCLP.^48^ Right UCLP was noted to have a stronger correlation with dental arch symmetry in a small Brazilian series.^49^ Dental arch relationships with sidedness of cleft were inconclusive; in the study by Staudt et al.,^50^ greater maxillary growth for left sided UCLP was reported using Modified Huddart Bodenham scores and overjet, whereas Haque et al.,^47^ reported no difference in GOSLON scores between left or right sided UCLP.

Educational attainment in reading was reported to be lower for left versus right UCL/P in a case control study performed in the USA.^51^ However, contradictory findings by the same author in a later paper, showed a greater need for academic support in school in right compared to left UCL/P involving a different study population.^22^ One hypothesis provided by the authors for the difference between the studies was that their first study excluded patients who had additional congenital anomalies. No difference in grade point averages at 16 years of age were noted among those with left versus right UCL/P in a Norwegian cohort study.^52^

A study published in German, which investigated functional outcomes including speech, reported no difference in intelligibility between left and right UCLP.^27^ Another study conducted in Japan investigated feeding and reported a lower proportion of tube feeding required for infants born with a left versus right UCL/P.^24^ A single study investigating psychological adjustment reported people born with right UCLP being more likely to request further surgical intervention compared to left UCLP.^53^

## Discussion

### Summary of Evidence

**Figure 2.**
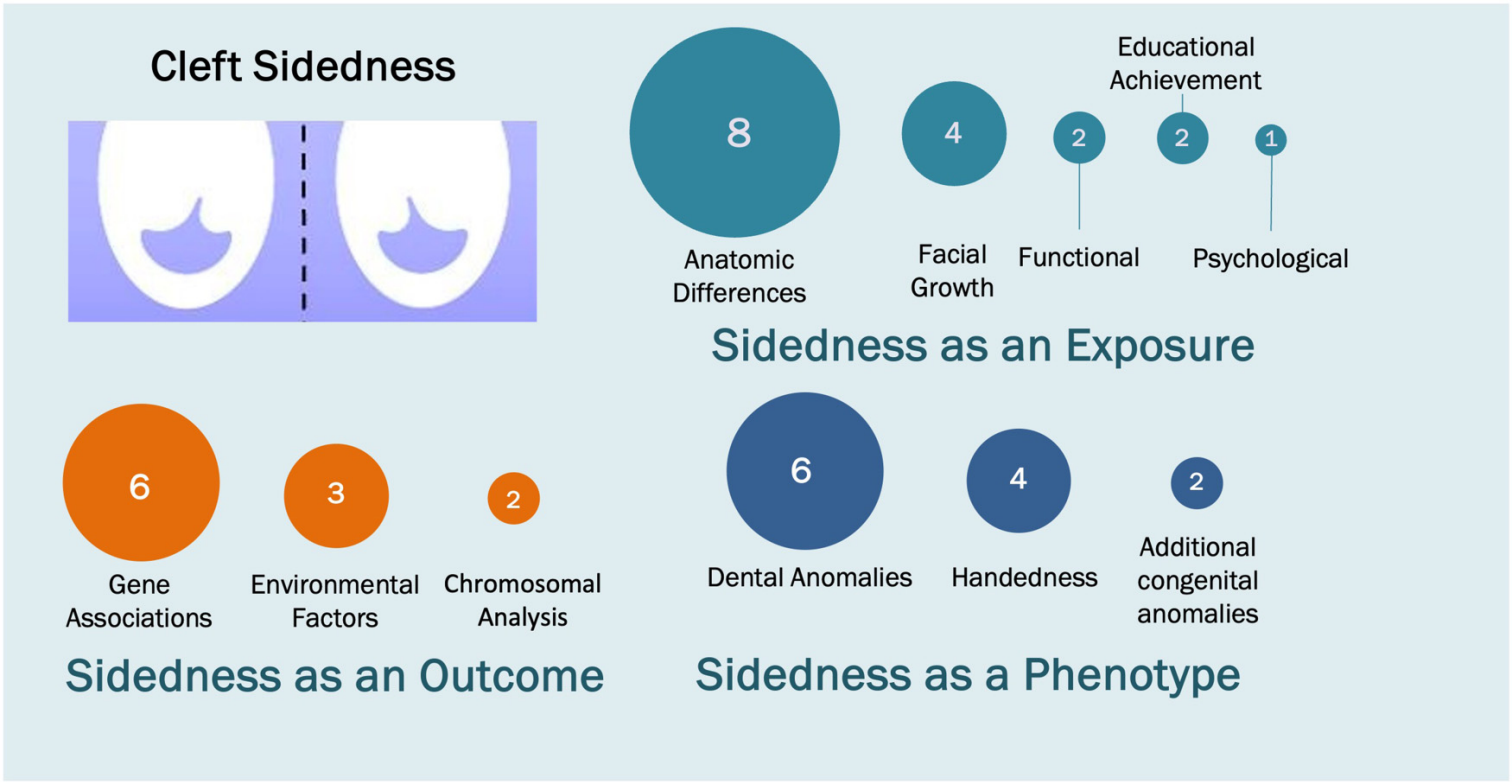
A mapping diagram to show the variables reported relating to sidedness in unilateral cleft lip with or without cleft palate.

The phenomenon of directional asymmetry in UCL/P with a 2:1 left to right ratio is universally accepted and established, yet despite this there has been relatively little research in the field of UCL/P sidedness as shown by the 40 studies that met our broad inclusion criteria in this scoping review (Figure 2). Studies reporting the co-occurrence of phenotypes with UCL/P sidedness spanned four decades, whereas studies focusing specifically on the cause, or outcomes of UCL/P sidedness were published more recently in the last three decades. In general, included studies were heterogeneous, covering a wide range of study designs, cleft subtypes and outcomes measures and whilst there were trends identified, inconsistencies in the results meant little consensus could be reached. We did not perform a quality assessment of the included studies but observe that more than half of the study designs (n = 21) were descriptive in nature (case studies, case series, and cross-sectional studies) with small sample sizes and for the most part without the adjustment for potential confounding factors.

### Strengths and Limitations

The strengths of this review include a comprehensive systematic search strategy with concerted efforts made to include all languages and a wide variety of study designs. This enabled inclusive mapping of the current literature in UCL/P sidedness. The main limitation of interpreting the results from the scoping review relate to the heterogeneity of the included studies. The broad inclusion of different study designs precluded a quality analysis, although we suspect the overall quality was low.

### Interpretation

Directional asymmetry in UCL/P is simple and straightforward in terms of its predictable preference for the left side in a 2:1 ratio. In all other respects, cleft sidedness is a fascinating yet complex phenomenon. Our review highlights the promising early findings in this field that has expanded steadily over the past four decades.

In terms of etiology, there was a precedent set in 1942 for cleft subtype differentiation by Fogh-Anderson, who demonstrated that cleft palate only is causally different from CL/P.^54^ It is now generally accepted that CL + -A, CLP and cleft palate only have distinct etiologies.^55–57^ Not only have studies within this review suggested it is plausible to consider distinct etiologies for left and right UCL/P but furthermore, the etiology of left versus right UCL + -A may be distinct from left versus right UCLP.^12,14,23,34^

Several theories have been postulated about the aetiology of directional asymmetry in UCL/P. The co-occurrence of left and right UCL/P with other phenotypes such as congenital dental anomalies, handedness and additional congenital anomalies are of interest because they could potentially have shared or linked aetiologies with UCL/P sidedness. The association between handedness and UCL/P sidedness has been an early focus in the literature because the mesoderm of the face is derived from the neural crest, therefore it is conceivable that there is a connection between cleft aetiology and cerebral hemisphere dominance. Preferential patterns of congenital dental anomalies according to UCL/P sidedness have also drawn interest in the literature because the development of tooth germs and the occurrence of cleft have close embryological relationships in terms of timing and anatomical position,^12^ but although patterns have been found, little conclusive evidence has been added to our overall understanding of laterality causality.

The association with other congenital anomalies is of interest because lip formation occurs embryologically at the same time as organogenesis.^5^ The consistent finding of right UCLP being associated with a higher prevalence of congenital anomalies in two studies suggests distinct embryological events between the formation of right and left orofacial clefts involving both the primary and secondary palate.^22,23^ Whilst asynchronous embryological fusions have indeed been described mainly in mouse models of embryological palate formation, it does not explain the consistent left sided preference.^58,59^

Proposed aetiological mechanisms involved in UCL/P sidedness have included specific genetic and environmental factors, hypoxia and the multifactorial threshold model. Discordant laterality of UCL/P in monogynous twins suggests that there is differential gene expression in the setting of an identical genotype.^60^ Differential gene expression has been shown to play an important role in the lateralization of the body during embryology^4^ and studies included in this review have suggested that the FAT4 and FGFR3 genes may be involved in cleft sidedness, but studies have been underpowered to conduct a full GWAS of left versus right UCL/P. The variety of genes and chromosomes implicated for both left and right UCL/P suggest a complex polygenic phenomenon, which fits with our current understanding of cleft aetiology. Further highly powered genetic studies are required to add clarity in this area.

The Multifactorial Threshold Model (MFTM) has been used to describe inheritance in orofacial cleft since the 1990s and involves numerous genes interacting in a multiplicative manner giving rise to an orofacial cleft occurring when the threshold is reached.^61^ The MFTM accepts a complex interplay of genetic and environmental factors but argues for a variable proportion of influence depending on the cleft subgroup. According to the model, right UCL/P is the less common trait and may represent a more severe phenotype, requiring a higher risk threshold and greater number of genetic and environmental factors.^51^ Right UCL/P has been suggested to be on a spectrum of phenotypic severity between a left sided UCL/P and a bilateral CL/P.^15,59^ The consistent finding of a higher prevalence of additional congenital anomalies in right UCLP in two studies in this review may lend support to the MFTM.^22,23^ The MFTM has been challenged in the literature^62^ and the level of evidence from this review cannot definitively substantiate, nor refute it.

In terms of outcomes impacted by UCL/P sidedness, our knowledge is limited by the relatively few publications in this field and the heterogeneity of outcomes studied. For anatomical differences in particular, a wide array of measurements were reported with only a minority showing differences between left and right UCL/P. Perhaps the subtle differences in outcomes between left and right UCL/P have failed to attract clinical research. Alternatively, the length of time needed to fully appreciate outcomes in cleft care may mean that few studies looking at long term outcomes are large enough to investigate this. Further work can now be focused on the measures that have been found to be different to help substantiate them.

We report a trend in results reported for either worse outcomes in right UCL/P or equivalent outcomes by UCL/P sidedness, with the proviso that a quality analysis was not performed. Anatomical and growth studies reporting worse outcomes for right sided UCL/P have reported a greater degree of hypoplasia in multiple tissue types compared to left sided UCL/P.^42,46,48,50^ Going forward it may help to advance our knowledge of outcomes in patients with UCL/P if awareness is raised amongst the community that sidedness in UCL/P could have a greater impact than purely in the administrative nomenclature of cleft classification.

### Summary and Recommendations

To summarize our findings according to our three initial objectives: first, this scoping review demonstrates an expanding evidence base in the field of UCL/P sidedness and included studies were all consistent in reporting a higher occurrence of left compared to right sided UCL/P in a 2:1 ratio. Second, studies focusing on aetiology of cleft sidedness have highlighted many examples to demonstrate different causes of left versus right UCL/P, although pathways remain poorly defined and understood. Third, there are trends for different outcomes when stratified by UCL/P sidedness, although these are currently inconsistent. These findings challenge previously held beliefs that UCL/P sidedness is simply an administrative feature for classification purposes alone and instead suggests that left and right UCL/P may have distinct etiologies and outcomes.

We make the following recommendations:

1. Awareness should be raised amongst cleft clinicians and researchers that left and right UCL/P should be formally considered as separate cleft subtypes in both clinical and research settings.
2. Furthermore, there is a suggestion that the cause (and therefore potentially the outcome) of UCL/P sidedness may be distinct between UCL + -A and UCLP subtypes. Sidedness should ideally be considered separately in research settings for UCL + -A and UCLP, as opposed to grouping together as UCL/P.
3. In order to investigate whether left and right sided UCL + -A or UCLP have different underlying genetic causes, adequately powered GWAS or DNA sequencing studies are needed which investigate left and right sided phenotypes as separate subgroups. Statistical methods could be used to investigate whether genetic variants play a role in the sidedness of cleft. One such method is PLACO (Pleiotropic analysis under composite null) which has previously been used to identify a locus in 1q32.2 (IRF6) that increases the risk of cleft palate only but decreases the risk for CL/P.^63^
4. Studies should be explicit about whether they include patients with additional congenital anomalies as the exclusion of patients is likely to skew the results of studies investigating UCL/P sidedness.
5. Confounding factors should be built into the analytic models to reduce the risk of bias. Sex should be given a high priority as a confounding factor in sidedness research as cleft subtypes are known to differ by sex, with UCLP being twice as common in males and cleft palate only being twice as common in females.^64^ Sex has been shown to influence the results as a co-variable in a study included in this review.^38^

## Supplemental Material

sj-docx-1-cpc-10.1177_10556656231221027 - Supplemental material for Sidedness in Unilateral Orofacial Clefts: A Systematic Scoping Review Supplemental material, sj-docx-1-cpc-10.1177_10556656231221027 for Sidedness in Unilateral Orofacial Clefts: A Systematic Scoping Review by Matthew Fell, Daniel Bradley, Ambika Chadha, Sophie Butterworth, Amy Davies, Craig Russell, Bruce Richard, Yvonne Wren, Sarah Lewis and David Chong in The Cleft Palate Craniofacial Journal

sj-docx-2-cpc-10.1177_10556656231221027 - Supplemental material for Sidedness in Unilateral Orofacial Clefts: A Systematic Scoping Review Supplemental material, sj-docx-2-cpc-10.1177_10556656231221027 for Sidedness in Unilateral Orofacial Clefts: A Systematic Scoping Review by Matthew Fell, Daniel Bradley, Ambika Chadha, Sophie Butterworth, Amy Davies, Craig Russell, Bruce Richard, Yvonne Wren, Sarah Lewis and David Chong in The Cleft Palate Craniofacial Journal

sj-docx-3-cpc-10.1177_10556656231221027 - Supplemental material for Sidedness in Unilateral Orofacial Clefts: A Systematic Scoping Review Supplemental material, sj-docx-3-cpc-10.1177_10556656231221027 for Sidedness in Unilateral Orofacial Clefts: A Systematic Scoping Review by Matthew Fell, Daniel Bradley, Ambika Chadha, Sophie Butterworth, Amy Davies, Craig Russell, Bruce Richard, Yvonne Wren, Sarah Lewis and David Chong in The Cleft Palate Craniofacial Journal
