# Supplementary material for: Sidedness in Unilateral Orofacial Clefts: A Systematic Scoping Review
Source: Cleft Palate Craniofac J. 2023 Dec 13;62(5):730–43. doi: 10.1177/10556656231221027 (PMC12106926; doi:10.1177/10556656231221027)
Supplement: sj-docx-2-cpc-10.1177_10556656231221027 - Supplemental material for Sidedness in Unilateral Orofacial Clefts: A Systematic Scoping Review [file sj-docx-2-cpc-10.1177_10556656231221027.docx]

| **Inclusion and Exclusion Criteria** | |
| --- | --- |
| **Include** | **Exclude** |
| **1. Publication:** Full-text papers published in a peer-reviewed journal. | **1. Publication:** Title, abstract or conference proceedings only or published in a non-peer reviewed journal (book, newspaper, or website). Grey literature. |
| **2. Study:** Studies which include individual patient data. This could include primary data of any study design including primary data from analytical (i.e. case-control, cohort) or descriptive studies (i.e. case reports, case series, cross sectional study) or secondary data (i.e. analysis of primary data such as systematic review). Animal studies will be included. | **2. Study:** Educational or editorial overviews, lacking individual patient data or animal data. |
| **3. Population:** Humans with unilateral clefts involving the embryological primary palate. This includes unilateral cleft lip +- alveolus +-cleft palate | **3. Population:** People or animals with bilateral cleft lip and palate or cleft palate only. |
| **4. Primary Outcome:** The outcome will be cleft laterality for studies of risk factors or mechanisms leading to cleft.  The outcome will be co-morbidities, anatomical differences and functional outcomes for studies where differences according to laterality are considered. | **4. Primary Outcome:**  Where laterality is only reported for locations remote to the cleft (ie brain laterality) rather than the cleft itself. |
