# Supplementary material for: Sidedness in Unilateral Orofacial Clefts: A Systematic Scoping Review
Source: Cleft Palate Craniofac J. 2023 Dec 13;62(5):730–43. doi: 10.1177/10556656231221027 (PMC12106926; doi:10.1177/10556656231221027)
Supplement: sj-docx-3-cpc-10.1177_10556656231221027 - Supplemental material for Sidedness in Unilateral Orofacial Clefts: A Systematic Scoping Review [file sj-docx-3-cpc-10.1177_10556656231221027.docx]

**Search Strategy**

**Embase <1980 to 2023 Week 21>**

[**https://ovidsp.ovid.com/ovidweb.cgi?T=JS&NEWS=N&PAGE=main&SHAREDSEARCHID=44cgQafwPDpdW56lyZi9xzPLJ6qiKTNPWucDmeUcih043H0W6R5Qdnp0DBrQzEzMB**](https://ovidsp.ovid.com/ovidweb.cgi?T=JS&NEWS=N&PAGE=main&SHAREDSEARCHID=44cgQafwPDpdW56lyZi9xzPLJ6qiKTNPWucDmeUcih043H0W6R5Qdnp0DBrQzEzMB)

1 cleft lip/ or "cleft lip with or without cleft palate"/ or bilateral cleft lip/ or unilateral cleft lip/ 15505

2 cleft lip*.tw. 16567

3 1 or 2 20910

4 hemispheric dominance/ 29642

5 (Lateralit* or Sided* or left* or right* or left side* or right side*).tw. 2984927

6 4 or 5 2994101

7 3 and 6 1773

**Ovid MEDLINE(R) <1946 to 2023 Week 21>**

<https://ovidsp.ovid.com/ovidweb.cgi?T=JS&NEWS=N&PAGE=main&SHAREDSEARCHID=1aAOY1wUENpdzQKQbQVlCLbYxoyurgwlnp44ofYaygEuBL0K2XdM8bJBBbUxZLosg>

1 Cleft Lip/ 16931

2 cleft lip*.tw. 14901

3 1 or 2 20322

4 dominance, cerebral/ or functional laterality/ 75080

5 (Lateralit* or Sided* or left* or right* or left side* or right side*).tw. 1195146

6 4 or 5 1234668

7 3 and 6 1017
